# Critical Involvement of Environmental Carbon Dioxide Fixation to Drive Wax Ester Fermentation in *Euglena*

**DOI:** 10.1371/journal.pone.0162827

**Published:** 2016-09-26

**Authors:** Adchara Padermshoke, Takumi Ogawa, Kazuki Nishio, Masami Nakazawa, Masatoshi Nakamoto, Atsushi Okazawa, Shigehiko Kanaya, Masanori Arita, Daisaku Ohta

**Affiliations:** 1 Graduate School of Life and Environmental Sciences, Osaka Prefecture University, Sakai, Osaka, Japan; 2 Graduate School of Information Science, Nara Institute of Science and Technology, Ikoma, Nara, Japan; 3 Center for Information Biology, National Institute of Genetics, Mishima, Shizuoka, Japan; 4 RIKEN Center for Sustainable Resource Science, Tsurumi, Kanagawa, Japan; National Renewable Energy Laboratory, UNITED STATES

## Abstract

Accumulation profiles of wax esters in *Euglena gracilis* Z were studied under several environmental conditions. The highest amount of total wax esters accumulated under hypoxia in the dark, and C28 (myristyl-myristate, C14:0-C14:0) was prevalent among all conditions investigated. The wax ester production was almost completely suppressed under anoxia in the light, and supplying exogenous inorganic carbon sources restored wax ester fermentation, indicating the need for external carbon sources for the wax ester fermentation. ^13^C-labeling experiments revealed specific isotopic enrichment in the odd-numbered fatty acids derived from wax esters, indicating that the exogenously-supplied CO_2_ was incorporated into wax esters via the propionyl-CoA pathway through the reverse tricarboxylic acid (TCA) cycle. The addition of 3-mercaptopicolinic acid, a phosphoenolpyruvate carboxykinase (PEPCK) inhibitor, significantly affected the incorporation of ^13^C into citrate and malate as the biosynthetic intermediates of the odd-numbered fatty acids, suggesting the involvement of PEPCK reaction to drive wax ester fermentation. Additionally, the ^13^C-enrichment pattern of succinate suggested that the CO_2_ assimilation might proceed through alternative pathways in addition to the PEPCK reaction. The current results indicate that the mechanisms of anoxic CO_2_ assimilation are an important target to reinforce wax ester fermentation in *Euglena*.

## Introduction

*Euglena gracilis* is a unicellular flagellated protist that possesses chloroplasts, which allow its autotrophic growth, and it can also grow as a heterotroph [1]. It is common in freshwater and can withstand harsh conditions that are harmful to microorganisms [2, 3]. When *Euglena* is grown aerobically in an organic carbon-rich medium, either in the light or in the dark, it stores appreciable amounts of the β-1,3-glucan, paramylon, as an energy reserve [4–8]. Transferring the aerobic culture to hypoxic conditions enables the synthesis of medium- to long-chain wax monoesters via a unique metabolic process called wax ester fermentation, along with the breakdown of the reserve paramylon. Switching the hypoxic culture back to aerobic conditions reverses the wax ester fermentation reactions [9]. The cellular adenosine triphosphate (ATP) content of *Euglena* decreases instantly upon exposure to hypoxic conditions, but gradually regains its original level when the wax ester fermentation becomes apparent and then stays constant during the anaerobiosis; thus, *Euglena* lives on respiration under normoxic conditions and on fermentation under hypoxic conditions [5].

The wax esters produced by *Euglena* under hypoxic culture consist exclusively of saturated carbon chains in both the fatty acid and fatty alcohol moieties, with myristyl myristate (C14:0-C14:0) as the major component [6, 7]. These neutral lipids are readily convertible into biofuels and thus they have received much attention as potential renewable feedstock for the production of petroleum substitutes [10, 11]. Because of the capability to synthesize large amounts of industrially-valuable wax esters from environmental CO_2_, *Euglena* is expected to be a competent microorganism to manage the growing greenhouse effects and global warming issues [10–14]. There are two main issues to be addressed when industrial application of *Euglena* is challenged. The first issue is the development of efficient pipelines for large-scale production of wax esters. Because the use of genetically modified microorganisms is not feasible in open-land cultivation, optimizing growth conditions of wild strains is a more favorable approach. The second issue is the quality manipulation of wax esters, which is primarily determined by the composition of fatty acid methyl esters (FAMEs) and fatty alcohols (FAs) [15]. Wax ester fermentation studies have thus far been performed using both wild strains and chloroplast-less auxotrophic *Euglena* mutants [16–24]. Tucci et al. have reported diverse potentials for wax ester accumulation levels and the FAME/FA composition among different *Euglena* strains under various O_2_ supply levels [8]. In this study we used the *E*. *gracilis* Z strain to investigate the culture conditions as a primary determinant of the wax ester accumulation levels rather than the modification of the wax ester compositions.

To clarify metabolic profiles during wax ester fermentation, we used gas chromatography-mass spectrometry (GC-MS) analysis of *E*. *gracilis* Z, a wild-type strain, cultured under a variety of environmental conditions. We showed that an external inorganic carbon source was essential for wax ester fermentation under anoxic conditions with continuous illumination. When *E*. *gracilis* Z was cultured in an anoxic environment, wax ester accumulation was reduced to nearly undetectable levels. The inhibited wax ester fermentation was recovered by supplementation of either CO_2_ gas or NaHCO_3_, suggesting the essential involvement of CO_2_ fixation that drives wax ester fermentation. Pönsgen-Schmidt et al. (1988) reported that phosphoenolypyruvate carboxykinase (PEPCK) is involved in CO_2_ fixation in photoautotrophic *Euglena* under dark anaerobic conditions [24]. Assuming PEPCK as a key enzyme for wax ester fermentation, we subjected *E*. *gracilis* Z culture to ^13^C-labeling experiments in the presence or absence of a PEPCK inhibitor (3-mercaptopicolinic acid; 3MPA). Here, we report that the externally applied ^13^CO_2_ was incorporated into wax esters via tricarboxylic acid (TCA) cycle intermediates. The time course of ^13^C-enrichment in these organic acids suggested that anaerobic ^13^CO_2_ fixation might proceed through multiple steps in the reverse TCA cycle, including PEP carboxylation and the carbon skeleton derived from α-ketoglutarate (α-KG). The current results indicate that reinforcement of anaerobic CO_2_ fixation ability offers a good opportunity for technology development toward efficient wax ester production in *Euglena*.

## Materials and Methods

### Cell culture and harvesting

*Euglena gracilis* wild-type Z strain cells were maintained aerobically in Koren-Hutner (KH) medium [25] in Sakaguchi flasks at 27°C under continuous light on a shaker (120 rpm) for 4 days (late-log phase) [26]. The pH of the fresh medium was adjusted to 5.0 with potassium hydroxide. For wax ester fermentation, the cells were maintained in 50 ml Erlenmeyer flasks at 28°C under continuous light (40 μmol m^-2^ s^-1^ photon flux density) with constant shaking (174 rpm) in an incubator shaker (BR-180LF, Taitec Co., Ltd., Saitama, Japan). The cells were cultured for 4 days to reach the late log phase (cell density of approximately 9 × 10^6^ cells ml^-1^) and subjected to different anaerobic treatments in a plant growth chamber (Biotron LPH200, Nippon Medical & Chemical Instruments, Co., Ltd., Osaka, Japan). All samples were harvested by centrifugation (1,600 × *g*, 25 sec, 4°C), then washed 3 times with 1 volume of pre-cooled 50 mM ammonium bicarbonate (NH_4_HCO_3_) (Nacalai Tesque Inc., Kyoto, Japan), and immediately frozen in liquid N_2_, freeze-dried and stored at -80°C until use.

### Wax ester fermentation

The wax ester fermentation in *E*. *gracilis* Z was investigated under either hypoxic or anoxic conditions. The late log phase cells were used for the wax ester fermentation study. Hypoxic cells were prepared by transferring an aliquot of 6 ml aerobic culture (the late-log phase) from the flask into a 15-ml conical sterile polypropylene centrifuge tube (Thermo Fisher Scientific, Inc., Waltham, MA, USA), which was then tightly capped and allowed to stand without agitation. Anoxic conditions were achieved by bubbling nitrogen (N_2_) gas (Taiyo Nippon Sanso, Co., Tokyo, Japan) into the aerobic culture for 10 min, thereby removing all the gaseous O_2_ and CO_2_ from the culture media. To study the effect of CO_2_ on wax ester fermentation, the anoxic culture was supplemented with either NaHCO_3_ (at either 10 or 20 mM final concentration), CO_2_ gas (10 min aeration; Taiyo Nippon Sanso, Japan) or CO_2_ gas followed by NaHCO_3_ (Nacalai Tesque, Japan) at various concentrations. All gases were passed through a Millex-FG filter unit (50 mm diameter, 0.20 μm, hydrophobic polytetrafluoroethylene; Merck Millipore Co., Billerica, MA, USA) before aeration to ensure sterility. For ^13^C-stable isotope labeling experiments, NaH^13^CO_3_ (Sigma-Aldrich Co., St. Louis, MO, USA) and ^13^CO_2_ gas (^13^C, 99%, WATARI, Co., Ltd., Kanagawa, Japan) were supplied as the ^13^C-labeled carbon sources. To monitor the changes in acidity and alkalinity of the culture medium induced by the anaerobic treatments, pH of the cultures was measured before and after anaerobiosis. For the systems treated solely by N_2_ aeration, a buffer, either 4-(2-hydroxyethyl)-1-piperazineethanesulfonic acid (HEPES, Gibco BRL, Bethesda, MD, USA) or *N*,*N*-bis-(2-hydroxyethyl)-2-aminoethanesulfonic acid (BES, Wako Pure Chemical Industries, Ltd., Osaka, Japan), was added up to 50 mM, and the medium pH fluctuations were within the range of 7.0 ± 0.5. Both the hypoxic and anoxic cultures were incubated at 28°C for 4 or 24 hours with or without illumination before harvesting for subsequent analyses. These current culture conditions (hypoxic-light and anoxic-light conditions in the presence or absence of external inorganic carbon supply) did not significantly affect growth, and oxygen levels in the culture medium were not noticeably different during the cultivation period of 4 hours or 24 hours. The time course of the ^13^C-labeling into organic acids were performed using the cells harvested at 5 min, 10 min, 20 min and 60 min after adding ^13^C. All samples were prepared in triplicate.

### Sample preparation and GC-MS analysis

Wax esters and organic acids were analyzed according to the methods described by Furuhashi et al. (2015) [26]. Briefly, approximately 5 mg of a lyophilized *E*. *gracilis* Z sample was homogenized in 1 ml of an extraction solvent comprising methanol, chloroform and 2% acetic acid (5:2:1, v/v/v) using a Mixer Mill (Retsch MM400, Verder Scientific Co. Ltd., Haan, Germany) for 90 s at 30 Hz. Testosterone (Sigma-Aldrich, USA) and ribitol (Nacalai Tesque, Japan) were used as internal standards when measuring nonpolar and polar metabolites, respectively. The homogenate was centrifuged (21,000 × *g*, 3 min, 20°C), and 400 μl ultra-pure water (Wako Pure Chemical Industries, Japan) and 500 μl chloroform (Sigma-Aldrich, USA) were added to 200 μl of the supernatant. The mixture was shaken vigorously and then centrifuged (21,000 × *g*, 3 min, 20°C) to separate the polar and nonpolar components. The bottom nonpolar phase and upper polar phase were collected separately, desiccated in a centrifugal evaporator (CC-105, Tomy Seiko Co. Ltd., Tokyo, Japan) and freeze-dried overnight. The pellets were incubated with 20 μl methoxyamine hydrochloride (Sigma-Aldrich)/pyridine (Merck Millipore, USA) solution (40 mg ml^-1^) at 30°C for 90 min, followed by silylation with 80 μl *N*-methyl-*N*-(trimethylsilyl)trifluoroacetamide (MSTFA, Sigma-Aldrich, USA) at 37°C for 30 min. GC-MS measurements were carried out on a gas chromatography-time of flight-mass spectrometer system (GC-TOF/MS) comprised of an auto sampler (PAL GC-xt, CTC Analytics AG, Zwingen, Switzerland), GC (Agilent 6890N, Agilent Technologies, Santa Clara, CA, USA) and MS (Micromass GCT Premier Mass Spectrometer, Waters Co., Milford, MA, USA). One microliter of the derivatized sample was injected into the GC-MS system using a cold trap splitless mode. The injection temperature was 230°C, and the flow rate of the carrier gas helium (>99.999% purity; Taiyo Nippon Sanso, Japan) was 1 ml min^-1^. The GC column was an HP-5MS capillary column (30 m × 0.25 mm × 0.25 μm; Agilent Technologies, USA). The following temperature program was applied: 70°C for 1 min, 1°C min^-1^ to 76°C, 6°C min^-1^ to 350°C, and 350°C for 1 min. The mass spectrometer was operated in an electron-impact (EI) mode at 70 eV within an *m*/*z* range of 40–600. The ion source temperatures were set at 320°C for wax ester analysis and 250°C for organic acid analysis. Metabolites were identified based on their mass spectral characteristics and retention times by comparison with retention times of reference compounds in an in-house reference library, and quantification was carried out using QuanLynx (Waters, USA). Student’s *t*-tests were performed using Microsoft Excel for Mac 2011 (Microsoft Co., Redmond, WA, USA).

## Results

### Wax ester fermentation in *E*. *gracilis* Z under hypoxia

Metabolic profiles of *Euglena* can be manipulated by controlling culture conditions. For example, paramylon and wax esters predominantly accumulate in normoxic-dark and hypoxic-dark cultures of *E*. *gracilis* Z, respectively, while proteins and other lipids are most abundant under normoxic-light conditions [27]. We studied the nature of the hypoxia-induced wax ester fermentation in *E*. *gracilis* Z under continuous illumination. After 4 days of normoxic-light growth, the culture was switched to hypoxia by stopping the shaking culture, and the cells were then kept in the light for additional 24 hours before they were harvested. The pH of the culture before and after the 24-h incubation was maintained in the range of 7.0 ± 0.5.

**Fig 1:**
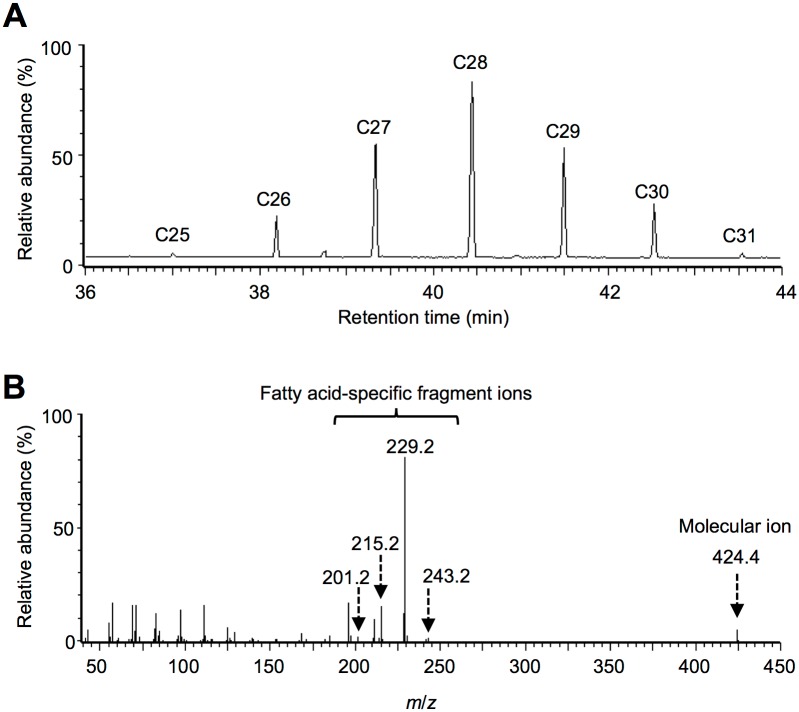
GC-MS analysis of the wax esters in *E*. *gracilis* Z under hypoxic-light conditions. (A) A total ion current (TIC) chromatogram from GC-MS analysis demonstrates wax ester peaks with the carbon chain lengths of C25, C26, C27, C28, C29, C30 and C31. (B) A mass spectral fragmentation pattern of the C28 wax ester displays parental ion (*m*/*z* 424.4) and fatty acid-specific fragment ions (*m*/*z* = 201.2, 215.2, 229.2, and 243.2 are attributable to C12:0, C13:0, C14:0 and C15:0 fatty acids, respectively).

While *E*. *gracilis* Z contains only very small amounts of wax esters under aerobic conditions, GC-MS analysis of the nonpolar fraction from the hypoxic samples indicated that wax esters were abundantly synthesized under hypoxic-dark conditions [26]. Fig 1 shows the gas chromatogram and representative mass spectrum of wax esters accumulated in *E*. *gracilis* Z under hypoxic-light conditions. The detected wax esters were identified by comparing their retention times and mass spectral fragmentation patterns with those of the authentic standard compounds [26]. As shown in Fig 1A, the majority of wax esters contained between 26 and 30 carbon atoms. The mass spectrum of the most abundant component (Fig 1B, C28 wax ester) exhibited a main fragment at *m*/*z* 229, attributable to C14:0 fatty acid, together with a molecular ion peak at *m*/*z* 424; this dominant species was identified as a C28 wax ester composed of myristic acid and myristyl alcohol (C14:0-C14:0). Other wax ester components were identified in a similar manner based on their retention times and mass spectral fragmentation patterns, and C14:0-C14:0 was shown to be the dominant wax ester in all samples [26]. The total chain lengths of wax esters synthesized under hypoxic-light conditions ranged from 22 to 34 carbon atoms, and unsaturated wax ester species were not detected. The wax ester accumulation profile in this study was in agreement with those reported in the previous study on the streptomycin-bleached mutant of *E*. *gracilis* Z [6, 26].

**Fig 2:**
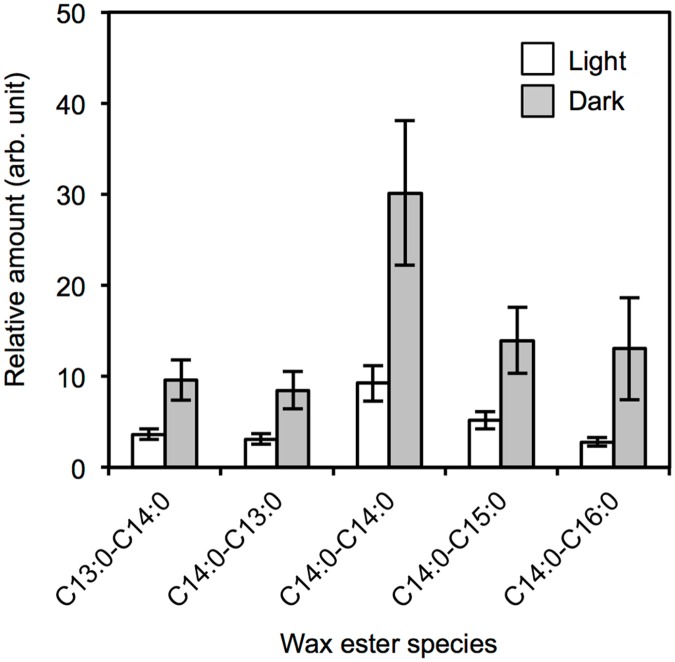
Major wax ester components in hypoxic *E*. *gracilis* Z cells. Light-grown cells in the late log phase were transferred to hypoxic culture by stopping the culture agitation. Error bars indicate standard deviation from triplicate cultures.

The wax ester fermentation in *Euglena* under photosynthetic conditions has rarely been addressed, presumably because the photosynthetic O_2_ generation could impede hypoxia and wax ester fermentation. Thus, we compared the accumulation profiles of wax esters between the light-grown and the dark-grown *E*. *gracilis* Z under the hypoxic conditions (Fig 2). The dark-grown cells accumulated larger amounts of wax esters than the light-grown cells. The decrease in the wax ester synthesis with increasing O_2_ concentration has been reported for *E*. *gracilis* SM-ZK and *E*. *gracilis* T [9, 24]. It is possible that the reduced levels of wax ester accumulation under the hypoxic-light conditions (Fig 2) may be due to a possible increase in O_2_ partial tension in the culture medium. The distribution of the five major wax esters (Figs 1 and 2), which altogether accounted for more than 70% of the total amounts synthesized under the hypoxic-light and hypoxic-dark conditions. Among all the samples, C14:0-C14:0 was most abundant followed by C14:0-C15:0, and the other three species existed in similar proportions (Fig 2). The similar wax ester profiles suggested that the light conditions mainly affected the amounts but not the varieties of lipid constituents of the wax esters lipid constituents.

### Wax ester fermentation in *E*. *gracilis* Z under anoxia

**Fig 3:**
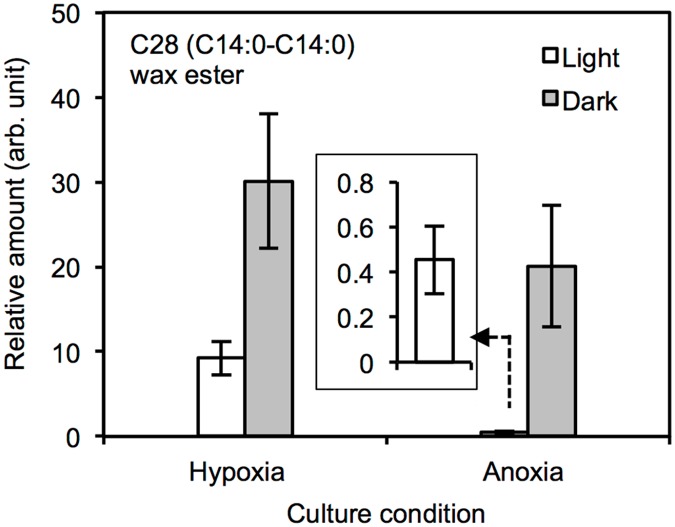
Inhibition of the wax ester fermentation in anoxia. The wax ester accumulation was almost completely abolished under anoxic-light conditions, as shown by the levels of C28 (14:0–14:0), which represents wax esters. The light-grown cells were subjected to anoxic and hypoxic conditions under both light and dark conditions. Hypoxia and anoxia were induced by stopping the culture agitation and N_2_ aeration (10 min), respectively. The results under light-anoxic conditions are magnified in the inset. Error bars indicate standard deviation from triplicate cultures.

We have, so far, studied wax ester fermentation in *E*. *gracilis* Z under hypoxic conditions initiated by stopping the shaking culture (Figs 1 and 2). To evaluate the metabolism under strictly anaerobic conditions, anoxic cells were prepared by bubbling N_2_ gas into the culture medium for 10 min. Then the cells were incubated either in the light or in the dark for additional 24 h before harvested. The pH of the culture medium before and after N_2_ aeration was 7.0 ± 0.5 and 7.7 ± 0.2, respectively. Despite fluctuations among experimental replicates after the 24-h incubation, the medium pH consistently increased to 9.0 ± 0.5 in the light, but stayed within the range of 7.0 ± 0.5 after dark incubation. Fig 3 shows the effects of light conditions on the accumulation levels of the dominant C28 wax ester, representing the general wax ester profile in *E*. *gracilis* Z, under both anoxic (N_2_ gas aeration) and hypoxic conditions (stopped shaking the culture). Irrespective of hypoxia and anoxia, the amount of wax ester accumulation was considerably higher in the dark-grown cells than the light-grown cells. We also observed a strikingly different result under anoxic-light conditions: the wax ester fermentation was nearly completely suppressed, while it was apparently unaffected in dark conditions (Fig 3). As described above, the culture medium pH increased during anoxic-light cultivation, where wax ester accumulation was greatly suppressed. The wax ester accumulation levels did not recover during 24-h of the anoxic-light, even when the initial pH 7.0 of the culture medium fluctuated within the range of pH 7.0 ± 0.5 in the presence of either HEPES or BES, suggesting that the inhibited wax ester accumulation was not simply a result of the increase in the medium pH.

The 10-min aeration with N_2_ gas to induce anoxia should have removed O_2_ and CO_2_ from the culture medium, and it can be assumed that CO_2_ deprivation may have negative effects on wax ester fermentation in light-grown *E*. *gracilis* Z. In other words, environmental CO_2_ is thought to be essential for wax ester fermentation. To address this issue, inorganic carbon sources, NaHCO_3_ and CO_2_ gas, were supplied to the *E*. *gracilis* Z N_2_-aerated culture in the light.

**Fig 4:**
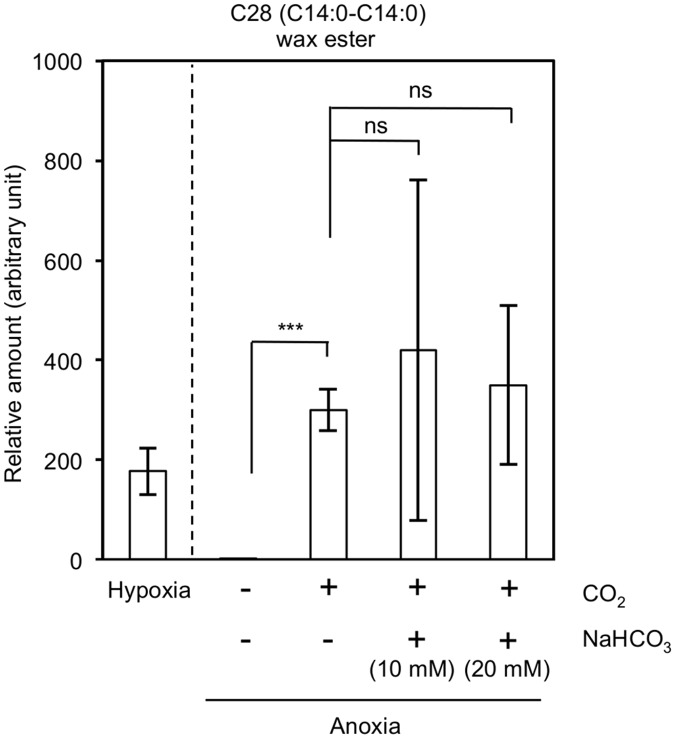
Recovery of the inhibited wax ester fermentation in anoxia by inorganic carbon sources. The relative levels of C28 (14:0–14:0) are shown as representing the wax esters in *E*. *gracilis* Z. The cells were cultivated in the light under hypoxic and anoxic conditions in the presence of CO_2_ and/or NaHCO_3_ (either 10 mM or 20 mM). The inset indicates light-anoxic conditions without an exogenous carbon supply. Error bars indicate standard deviation from triplicate cultures.

Fig 4 compares the relative accumulation levels of C28 wax ester, representing the general wax ester profile in *E*. *gracilis* Z, under various conditions. C14:0-C14:0 wax ester was most abundant in all the samples investigated. Supplementing CO_2_ gas restored wax ester fermentation in light-grown anoxic *E*. *gracilis* Z cells (Fig 4). Supplementation of NaHCO_3_ (either 10 mM or 20 mM) to the CO_2_-aerated sample did not have additional influence on the wax ester accumulation (Fig 4). The amounts of wax esters accumulated in the CO_2_-aerated anoxic cells were slightly higher than that produced in the hypoxic culture (Fig 4). Distribution of the five major wax esters synthesized under these growth conditions is shown in Table 1.

**Table 1:** Accumulation profiles of the five major wax ester species in *E*. *gracilis* Z under hypoxic, anoxic + CO_2_, and anoxic + CO_2_ + NaHCO_3_ conditions.

Wax esters	Percentage (%)							
	Hypoxia		Anoxia + CO_2_		Anoxia + CO_2_ + NaHCO_3_ (10 mM)		Anoxia + CO_2_ + NaHCO_3_ (20 mM)	
	Average	SD	Average	SD	Average	SD	Average	SD
C13:0-C14:0	11	0.7	5	0.3	6	0.8	9	3.5
C14:0-C13:0	9	0.4	5	0.3	6	0.7	8	2.9
C14:0-C14:0	37	2.0	46	4.0	46	3.0	38	9.0
C14:0-C15:0	15	1.0	14	2.0	14	2.0	17	3.0
C14:0-C16:0	5	0.5	12	1.0	11	1.0	8	4.9
Others	24	0.2	18	0.1	18	0.1	22	0.5
Total amount (% dry weight)	14	2.2	18	2.9	24	17.8	24	8.1

The light-grown cells in the late log phase were transferred to hypoxic and anoxic conditions by stopping the culture agitation and N_2_ aeration (10 min), respectively. All data are the average of triplicate culture experiments.

These results demonstrated that external carbon sources are required for wax ester biosynthesis under anoxic light conditions. Thus, the reserve paramylon or its derivative carbohydrates were not readily usable under anoxic-light conditions without external carbon sources. Under anoxic-light conditions, addition of NaHCO_3_ slightly promoted the wax ester fermentation [26], which was fully restored by supplying CO_2_ gas into the culture medium (Fig 4). At pH 6.3 ± 0.1, 41–47% of the dissolved carbon in the culture medium should be in the form of HCO_3_^-^ [28], suggesting the presence of unidentified routes to favor CO_2_ over HCO_3_^-^. In addition, wax ester fermentation under anoxic-dark conditions proceeded even when there was a shortage of exogenous CO_2_ (Fig 3), suggesting the presence of a distinct metabolic system that operates in the dark for wax ester fermentation.

### Anoxic ^13^CO_2_ assimilation

We demonstrated that the presence of external inorganic carbon sources was essential for the wax ester fermentation. To clarify the metabolic role of external CO_2_, we first performed ^13^C-labeling experiments using NaH^13^CO_3_ during wax ester fermentation under anoxia. After the simultaneous aeration of N_2_ and CO_2_, the anoxic culture was supplemented with NaH^13^CO_3_ (20 mM) and incubated in the light for 4 h or 24 h before it was harvested. The pH of the culture was about 6.7 ± 0.1, and thus 64–69% HCO_3_^-^ and 36–31% CO_2_ should be in equilibrium [28]. Table 2 compares the ^13^C-isotopomer incorporation levels among the wax esters with the carbon chain length ranging from C26 to C30 produced during 4 h and 24 h cultivation periods. The cells were cultured under hypoxic-light conditions (control group) and anoxic-light conditions using CO_2_ gas and NaH^13^CO_3_ (20 mM) supplementation (test group). The data were derived from the comparison of mass spectral intensities of the natural isotope (M) and the ^13^C-labeled (M+1) fragments of the fatty acids that are characteristic to each of the wax ester species. For example, mass spectral intensities of C14:0 fatty acid fragments (M; *m/z* = 229, M+1; *m/z* 230) were used to calculate the percentage of ^13^C-isotopomer for C14:0-C14:0 wax ester. The Student’s *t*-test was used to evaluate whether or not the ^13^C-enrichment levels were different between odd-numbered fatty acids and even-numbered fatty acids. For the 4 h cultivation experiment, data were only available for the C27 and C28 wax esters that had sufficiently high abundance for the calculation of ^13^C-enrichment. Compared with the control group, ^13^C-labeled samples showed higher percentages of the ^13^C-isotopomer in the C13:0 fatty acid fragments from C26, C27, C28 and C29 wax esters and in the C15:0 fatty acid fragment from the C29 and C30 species. All even-numbered fatty acids, i.e., C12:0, C14:0 and C16:0, however, did not show a significant difference between the control and test groups. This labeling pattern was also observed with the 4-h incubated culture. These observations confirmed that the ^13^C-labeled inorganic carbon sources are incorporated into the wax esters and they are also required to drive wax ester fermentation. The isotopic enrichment observed in this experiment was relatively low (Table 2), probably reflecting the fact that CO_2_ rather than HCO_3_^-^ is the preferred carbon source for wax ester fermentation. It has been reported that different *Euglena* strains were characterized by diverse wax ester fermentation abilities, and that the relative proportions of wax ester components, FAMEs and FAs, were modifiable by changing the oxygen levels [8]. The current results indicate that the wax ester compositions in *E*. *gracilis* Z did not change under different culture conditions (Tables 1 and 2).

**Table 2:** Incorporation of externally-added ^13^C into wax esters in *E*. *gracilis* Z.

Wax esters	Fatty acid fragments	M+1/M (%)							
		^13^C incorporation period of 4 h				^13^C incorporation period of 24 h			
		Control group	Test group	*P*-value		Control group	Test group	*P*-value	
C26	C12:0	ND	ND	-		2.11	2.57	1.63 E-03	**
	C13:0	ND	ND	-		2.26	5.64	7.26 E-10	***
	C14:0	ND	ND	-		2.20	2.68	1.59 E-02	*
C27	C12:0	ND	ND	-		1.66	1.82	3.60 E-01	
	C13:0	2.17	4.43	2.21 E-06	***	2.60	6.24	1.24 E-09	***
	C14:0	2.11	ND	-		2.59	3.27	6.74 E-02	
C28	C12:0	ND	ND	-		1.44	1.33	4.94 E-01	
	C13:0	2.18	4.52	1.21 E-02	*	2.12	4.26	2.12 E-06	***
	C14:0	2.56	2.41	4.76 E-01		3.13	3.42	1.68 E-01	
C29	C13:0	ND	ND	-		2.07	5.51	2.39 E-09	***
	C14:0	2.36	ND	-		3.04	3.37	3.05 E-01	
	C15:0	ND	ND	-		1.97	4.67	1.38 E-08	***
C30	C14:0	ND	ND	-		2.70	3.45	1.98 E-02	*
	C15:0	ND	ND	-		2.25	4.91	6.08 E-07	***
	C16:0	ND	ND	-		2.65	2.60	7.64 E-01	

Light-grown cells in late log phase underwent a ^13^C incorporation period of 4 h and 24 h under light and under hypoxic conditions (control group) and anoxic conditions in the presence of CO_2_ supplemented with NaH^13^CO_3_ (20 mM) (^13^C-labeled test group). The ratios of M+1 (^13^C isotopomer) to M (^12^C) are obtained from characteristic fatty acid fragments from wax esters. Values are presented as the mean ± standard deviation from triplicate analyses from triplicate cultures. Asterisks indicate statistically significant differences (Student’s *t*-test, *p < 0.05, **p < 0.01, ***p < 0.001); ND, not detected.

### Clarification of the anoxic ^13^C incorporation steps

The ^13^C enrichment in odd-numbered fatty acids under anoxic-light conditions (Table 2) is consistent with the possibility that ^13^C must have been incorporated into wax esters via the propionyl-CoA pathway [8, 29]. Considering the early metabolic steps leading to wax ester fermentation via the propionyl-CoA pathway, PEPCK is the most plausible candidate enzyme responsible for the CO_2_ fixation. To verify this possibility, we performed ^13^C-labeling experiments in the presence or absence of 3MPA, an inhibitor of PEPCK [30]. To ensure higher sensitivity to monitor ^13^C-enrichment, we used ^13^CO_2_ gas, which was shown to be a preferential form of organic carbon sources for wax ester fermentation (Fig 4). Additionally, we modified the KH medium composition [25]. Although the KH medium is the most common medium for *Euglena* culture, it is comprised of 66 mM glucose, 48 mM malate, 2 mM citrate and 0.6 mM succinate together with 3 mM NH_4_HCO_3_, 7 mM MgCO_3_ and 2 mM CaCO_3_. These constituents should inevitably influence the sensitivity of the ^13^C-labeling experiment. The modified KH medium was prepared by excluding malate, succinate, citrate, NH_4_HCO_3_, MgCO_3_ and CaCO_3_. *E*. *gracilis* Z growth in the modified KH medium under light conditions for at least during 24 h was not apparently different from that observed in the KH medium.

**Fig 5:**
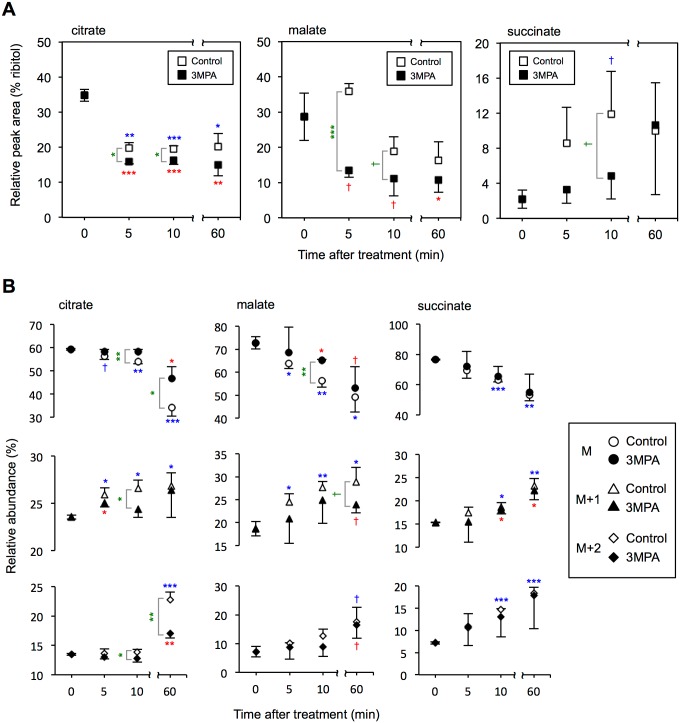
^13^C-labeling time course of organic acids in the presence or absence of 3MPA. (A) Relative peak areas of organic acids in the presence (black symbol) or absence (white symbol) of 3MPA. (B) Relative abundances of M, M+1 and M+2 ions characteristic to each organic acid in the presence (3MPA-treated cells, black symbol) or absence (control cells, white symbol) of 3MPA. Values and error bars are the mean and standard deviation from triplicate cultures. Asterisks and daggers indicate statistically significant differences. Red (3MPA treatment) and blue (control) indicate statistically significant differences between 0 min and respective time points for each treatment. Student’s *t*-test with Bonferroni correction; statistical significance between treatments at each time point (green); Student’s *t*-test; † *p* < 0.10, *p < 0.05, **p < 0.01, ***p < 0.001.

Fig 5 shows the time course experiments to monitor the CO_2_ assimilation under the anoxic-light condition. Cells in the modified KH medium were supplied with ^13^CO_2_ gas after 10 min of N_2_ gas aeration. Oxaloacetate (OAA) and fumarate were not detectable under our experimental conditions. Under anoxic-light conditions, the citrate levels started to decline within 5 min of supplying ^13^CO_2_ and it reached about 57% (without 3MPA, control cells) and 48% (3MPA-treated cells) of the initial level at 60 min, and were consistently higher in the control cells during the period (Fig 5A). The malate levels also decreased and reached about 62% (control) and 41% (3MPA-treated cells) of the initial level during 60 min (Fig 5A). A continuous decrease in the citrate and malate levels (Fig 5A), irrespective of 3MPA treatment, indicated that the pre-accumulated metabolite pools in the normal KH medium were consumed during wax ester fermentation in the modified KH medium. However, succinate levels continued to be elevated during the experiments irrespective of the 3MPA treatments (Fig 5A). The natural isotope (M) abundances (Fig 5B) suggested that the 3MPA treatment inhibited consumption of the pre-accumulated citrate and malate metabolic pools and that succinate metabolism was apparently unaffected by 3MPA.

Both M+1 and M+2 citrate levels started to increase within 5 min after supplying ^13^CO_2_, while the levels were consistently lower in 3MPA-treated cells during the time course (Fig 5B). The M+1 malate isotope ion levels were elevated quickly and it remained elevated after supplying ^13^CO_2_, while the presence of 3MPA was inhibitory toward ^13^C-enrichment in malate, which supports ^13^CO_2_ incorporation into malate via OAA production by the PEPCK reaction (Fig 5B). However, the abundance of both M+1 and M+2 in succinate were similarly elevated in both treatments, suggesting the presence of succinate production routes that are independent of the 3MPA-sensitive PEPCK reaction (Fig 5B).

## Discussion

### ^13^C-labeling into odd-numbered fatty acid moieties

**Fig 6:**
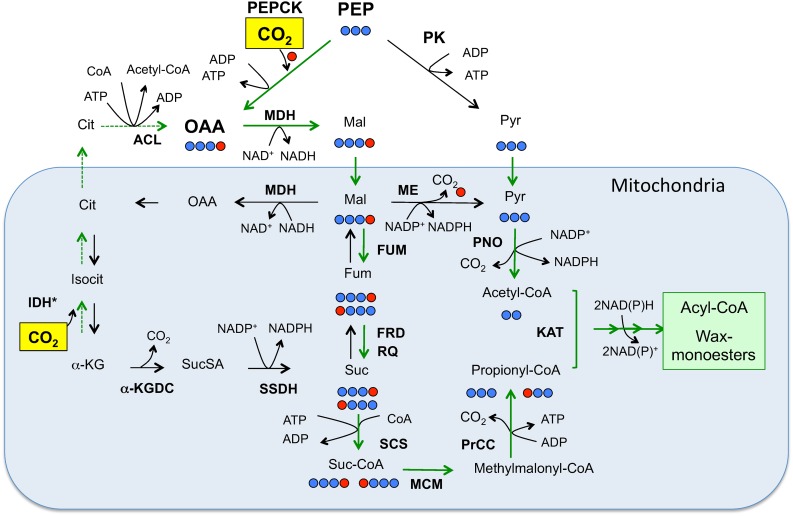
Wax ester fermentation in *E*. *gracilis*. Under anaerobic conditions, mitochondrial wax ester fermentation proceeds through two routes: the C2-donor route supplies acetyl-CoA via the pyruvate:NADP^+^ oxidoreductase (PNO) reaction, and the C3-donor route includes anaerobic fumarate respiration to produce propionyl-CoA. The ^13^C-isotope from ^13^CO_2_ (red circle) is incorporated into phosphoenolpyruvate (PEP) by the PEPCK reaction, and it is retained in propionyl-CoA that is used as the C3-donor in wax ester fermentation. The metabolic flow functioning under anaerobic conditions is indicated by green arrows [8, 15, 29, 31]. Another possible route for the carboxylation reaction is indicated by broken green arrows: α-ketoglutarate (α-KG) is carboxylated to yield citrate which ultimately serves as the precursor to produce oxaloacetate (OAA) in cytoplasm. **Abbreviations for key enzymes:** ACL, ATP citrate lyase; FUM, fumarase; FRD, fumarate reductase; IDH*, isocitrate dehydrogenase; KAT, ketoacyl-CoA thiolase; α-KGDH, α-ketoglutarate decarboxylase; MDH, malate dehydrogenase; ME, malic enzyme; MMC, methylmalonyl-CoA mutase; PK, pyruvate kinase; PNO, pyruvate:NADP^+^ oxidoreductase; PrCC, propionyl-CoA carboxylase; SSDH, succynyl semialdehyde dehydrogenase; RQ, rhodoquinone; SCS, succinyl-CoA synthetase. Abbreviations for metabolic intermediates: Cit, citrate; Fum, fumarate; Isocit, isocitorate; Mal, malate; OAA, oxaloacetate; Pyr, pyruvate; Suc, succinate; SucSA, succinate semialdehyde. *IDH has not been known in *Euglena* to produce isocitrate from α-KG.

To clarify the essential role of inorganic carbon source assimilation, we performed ^13^CO_2_-feeding experiments and demonstrated that the ^13^C-stable isotope was primarily incorporated into wax ester odd-numbered fatty acid moieties. This is consistent with the findings from ^14^C-radioisotope-labeling experiments. Schneider and Betz [23] reported that H^14^CO_3_^-^ as well as [1,4-^14^C] succinate and [3-^14^C] propionate were preferentially incorporated into odd-numbered fatty acids and alcohols in anaerobically-grown *E*. *gracilis* T under dark conditions. In addition, it has been reported that [2-^14^C] pyruvate was not anaerobically incorporated into succinate, indicating that pyruvate carboxylase might not be primarily involved in the CO_2_ fixation into wax esters via OAA production [23]. Instead, the considerable decrease in 2-phosphoglycerate, pyruvate and PEP levels in fermenting *E*. *gracilis* Z under anaerobic-dark conditions [27] suggests the involvement of PEP in wax ester biosynthesis. Thus, upon incorporation of CO_2_ as shown in Fig 6, PEP is converted to OAA, which subsequently undergoes the reverse TCA cycle to yield succinyl-CoA and finally propionyl-CoA via the methylmalonyl-CoA intermediate [23]. Additionally, in the reverse TCA cycle, α-KG could be carboxylated to give isocitrate, which serves as a TCA cycle intermediate (Fig 6). However, α-KG carboxylation involved in wax ester fermentation has not been demonstrated in *Euglena*.

### CO_2_ assimilation and wax ester fermentation

The essential role of environmental CO_2_ fixation to drive wax ester fermentation, particularly via biosynthesis of odd-numbered fatty acids, could be ascribed to mitochondrial energy metabolism under anaerobic condition. Thus, the OAA produced by PEPCK is converted to malate to fuel the anaerobic fumarate reductase system to produce succinate (Fig 6). Rhodoquinone-dependent fumarate reductase is responsible for the conversion of fumarate to succinate, and the electron transfer to supply the reduced form of rhodoquinone is linked to mitochondrial H^+^ transport leading to ATP synthesis [29, 31]. It has been reported that anaerobically-grown *Euglena* cells contain rhodoquinone at higher levels than aerobically-grown cells [32], and Castro-Guerrero et al. (2005) proposed an electron transfer chain in *Euglena* containing D-lactate dehydrogenase through rhodoquinone, which supports fumarate reductase activity under anaerobic conditions [33].

### Anaerobic CO_2_ assimilation steps

In general, two enzymes, PEP carboxylase and PEPCK, are responsible for the carboxylation of PEP to produce OAA. PEP carboxylase is thought to be involved in anaplerotic CO_2_ fixation in the dark, which is linked to amino acid metabolism [22], while it is likely that PEPCK (Fig 6) is primarily involved in the anaerobic CO_2_ fixation [24]. The metabolic process involving OAA and malate constitutes part of the reverse TCA cycle, where OAA is eventually reduced to malate to give succinate via the fumarase reaction. In this reverse TCA cycle, the assimilated ^13^CO_2_ is retained within the succinate carbon skeleton, and then incorporated into odd-numbered fatty acids (Fig 6). When autotrophically maintained *E*. *gracili*s T was supplemented with glucose and CO_2_, PEPCK activity was strongly induced [24], and the induction levels were much higher in the dark than that in the light [24]. PEPCK in *Euglena* is ADP- or guanosine diphosphate (GDP)-dependent [8, 23].

The ^13^C-enrichment in succinate was apparently unaffected by the 3MPA treatment, suggesting that there are alternative ^13^CO_2_ routes of assimilation leading to succinate production. As described above, a possible route for ^13^CO_2_ incorporation into succinate may be through reductive carboxylation of α-KG in the reverse TCA cycle to produce isocitrate (Fig 6). In hypoxic cancer cells, glutamine-derived α-KG is carboxylated by a mitochondrial isoform of isocitrate dehydrogenase to produce isocitrate, which consumes NADH [34–36]. Mitochondrial aconitase can then convert isocitrate to citrate [37], which serves as the substrate for cytosolic ATP-citrate lyase to generate oxaloacetate and acetyl-CoA [38]. In this metabolic route (Fig 6), CO_2_ incorporated into α-KG is retained within the oxaloacetate carbon skeleton, which could be transported back to mitochondria, and the acetyl-CoA is used in the biosynthesis of fatty acids and cholesterol. In this metabolic route, the incorporated ^13^CO_2_ will be retrieved into succinate. However, in *Euglena*, the involvement of mitochondrial isocitrate dehydrogenase and cytosolic ATP-citrate lyase in reductive CO_2_ fixation is not fully understood.

## Conclusion

We have demonstrated that *E*. *gracilis* Z was unable to accomplish wax ester fermentation in the absence of external inorganic carbon sources under light-anoxic conditions. When *E*. *gracilis* Z was subjected to wax ester fermentation in the presence of H^13^CO_3_^-^, the ^13^C-isotope was assimilated into wax esters. Thus, assimilation of environmental CO_2_ is a critical step to drive wax ester fermentation and it is also linked to the production of building blocks for wax esters. Environmental CO_2_ could be incorporated into wax esters via the PEPCK reaction and the metabolic link involving α-KG and glutamate/glutamine in the reverse TCA cycle. The current results indicate that engineering an efficient substrate supply to fuel anoxic CO_2_ fixation is a future challenge toward increasing wax ester production.
